# Growth Substrate and Prophage Induction Collectively Influence Metabolite and Lipid Profiles in a Marine Bacterium

**DOI:** 10.1128/msystems.00585-22

**Published:** 2022-08-16

**Authors:** Jonelle T. R. Basso, Katarina A. Jones, Kaylee R. Jacobs, Courtney J. Christopher, Haley B. Fielland, Shawn R. Campagna, Alison Buchan

**Affiliations:** a Department of Microbiology, University of Tennessee Knoxville, Knoxville, Tennessee, USA; b Department of Chemistry, University of Tennessee Knoxville, Knoxville, Tennessee, USA; c Biological and Small Molecule Mass Spectrometry Core, University of Tennessee Knoxville, Knoxville, Tennessee, USA; University of Copenhagen

**Keywords:** *Roseobacter*, temperate phage, growth conditions, metabolomics, lipidomics, bacterial physiology

## Abstract

Bacterial growth substrates influence a variety of biological functions, including the biosynthesis and regulation of lipid intermediates. The extent of this rewiring is not well understood nor has it been considered in the context of virally infected cells. Here, we used a one-host-two-temperate phage model system to probe the combined influence of growth substrate and phage infection on host carbon and lipid metabolism. Using untargeted metabolomics and lipidomics, we reported the detection of a suite of metabolites and lipid classes for two *Sulfitobacter* lysogens provided with three growth substrates of differing complexity and nutrient composition (yeast extract/tryptone [complex], glutamate and acetate). The growth medium led to dramatic differences in the detectable intracellular metabolites, with only 15% of 175 measured metabolites showing overlap across the three growth substrates. Between-strain differences were most evident in the cultures grown on acetate, followed by glutamate then complex medium. Lipid distribution profiles were also distinct between cultures grown on different substrates as well as between the two lysogens grown in the same medium. Five phospholipids, three aminolipid, and one class of unknown lipid-like features were identified. Most (≥94%) of these 75 lipids were quantifiable in all samples. Metabolite and lipid profiles were strongly determined by growth medium composition and modestly by strain type. Because fluctuations in availability and form of carbon substrates and nutrients, as well as virus pressure, are common features of natural systems, the influence of these intersecting factors will undoubtedly be imprinted in the metabolome and lipidome of resident bacteria.

**IMPORTANCE** Community-level metabolomics approaches are increasingly used to characterize natural microbial populations. These approaches typically depend upon temporal snapshots from which the status and function of communities are often inferred. Such inferences are typically drawn from lab-based studies of select model organisms raised under limited growth conditions. To better interpret community-level data, the extent to which ecologically relevant bacteria demonstrate metabolic flexibility requires elucidation. Herein, we used an environmentally relevant model heterotrophic marine bacterium to assess the relationship between growth determinants and metabolome. We also aimed to assess the contribution of phage activity to the host metabolome. Striking differences in primary metabolite and lipid profiles appeared to be driven primarily by growth regime and, secondarily, by phage type. These findings demonstrated the malleable nature of metabolomes and lipidomes and lay the foundation for future studies that relate cellular composition with function in complex environmental microbial communities.

## INTRODUCTION

The macromolecular composition of bacterial cells is the product of both external and internal factors. The concentrations and chemical forms of carbon and nutrients influence bacterial physiology in a strain-specific fashion. For instance, growth substrates affect cellular growth rates, size, morphology, and other dynamic biological functions (1). The processing of growth substrates through central metabolism (e.g., the tricarboxylic acid [TCA] cycle) influences the synthesis and regulation of many important macromolecules, including key lipid biosynthesis intermediates (1, 2). However, the extent to which substrates and nutrients utilized for growth are directly related to cell lipid composition is not fully understood. This knowledge gap presents challenges to the application of lab-based studies of isolates to the interpretation of field-based measurements of natural microbial populations.

Lipids are key constituents of bacterial cell membranes and are organized into a few broad classes, including the common phospholipids phosphatidylethanolamine (PE), phosphatidylglycerol (PG), cardiolipin (CL), phosphatidylcholine (PC), phosphatidylinositol (PI) and phosphatidylserine (PS) (3–6). Despite the diversity of phospholipid head groups recognized among bacteria, a single key precursor to all of these molecules is phosphatidic acid (PA) (7). Other lipid classes, such as aminolipids and other phosphorus-free lipids (e.g., sulfoquinovosyldiacylglycerol [SQDG]) have been documented in diverse bacteria where they have been linked to environmental stress, most notably phosphorus limitation ((8); reviewed in reference (9, 10)). Indeed, cellular lipid composition can be viewed as an adaptive response. It is nonstatic and favors forms that provide the necessary structural features for cells under specified environmental conditions (9). Cellular modulation of lipid composition has been linked to temperature and pH as well as production and accumulation of metabolites and nutrient levels (reviewed in references (9, 11)). Because lipid biosynthesis is intrinsically linked to central metabolism, factors influencing central metabolism, including viral infection, may also be expected to affect lipid composition.

Phage-dependent manipulation of bacterial host metabolic processes can occur through redirection of pathway-specific mechanisms, including alterations in central carbon metabolism, nucleotide, and/or lipid biosynthesis pathways (reviewed in references (12–16)). In turn, central metabolism can influence phage infection. For example, growth substrate has been shown to influence prophage induction (e.g., (17, 18)), indicating that the host metabolic state can have a direct influence on the lysogenic-lytic decision. Direct linkages between phage infection and host lipid composition provide further insight into the varied fitness strategies employed by viruses. For instance, cyanophage-encoded auxiliary metabolic genes (AMGs), specifically fatty acid desaturases, have been implicated in alterations to host cyanobacterial membrane fluidity, which are suggested to lead to host photoprotection (19). While AMGs have been involved in virus-mediated host metabolic remodeling, and their identification can lead researchers to specific, targeted pathways, the presence of such genes does not appear to be a requirement for metabolic redirection. Furthermore, in the absence of obvious AMGs, such redirections are not easily predictable from viral genetic signatures alone (20, 21).

Community-level metabolomics approaches are increasingly used to characterize natural microbial populations (22). Current techniques are necessarily applied to discrete samples representing ‘snap shots’ of dynamic communities from which the status and function of these communities, or selected members within, are then inferred (e.g., (23–25)). To facilitate the interpretation of community-level data, the extent to which ecologically relevant bacteria demonstrate metabolome flexibility requires elucidation. Herein, we used an environmentally relevant heterotrophic marine bacterium of the *Sulfitobacter* genus to assess the influence of growth medium on primary metabolite and lipid composition. This bacterium is a representative of the *Roseobacter* lineage, a dominant marine lineage whose members are active and abundant components of microbial assemblages in diverse marine niches (26). Like most bacteria characterized to date (e.g., (27, 28)), this strain harbored a prophage. We also had a closely related strain that harbored a genetically similar prophage but demonstrated a high rate of spontaneous prophage induction, that is activation of the lytic cycle in the absence of obvious stressors (29). These strains were analyzed in parallel to gain insight into the influence of prophage induction, a common phenomenon in marine systems (reviewed in reference (30)), on fundamental cellular processes.

## RESULTS

The *Sulfitobacter* strains CB-A and CB-D utilized in this study are lysogenized with a single temperate phage, Φ-A prophage or Φ-D prophage (here prophage-A or prophage-D, respectively). These prophages shared an integration site in their host but are incompatible within a single host. That is, only a single prophage genotype could be stably maintained in this host strain. In addition, each lysogenized strain is susceptible to infection by the alternate phage genotype (29). CB-A was derived from an infection of CB-D with ΦA virions: prophage-A effectively replaced the original prophage-D. The two prophages are integrated into the same site in both strains and share 79% nucleotide identity (31, 32). The sequence variation between the two phages was localized to two 3 to 4 kb regions which primarily encode genes of unknown function, but also (i) tail fibers proteins, expected to be important for binding to host cell surface receptors (33) and (ii) transcriptional regulators that may be involved in repression of lytic genes during lysogeny. The putative transcriptional regulators encoded within each phage bear little sequence homology to one another. Sequence identity at the amino acid level was <30% for all pairwise alignments. ΦD harbored 2 open reading frames (ORFs) (SUFP_003; SUFP_050) that fell within the XRE transcriptional regulator superfamily. Both contained an XRE-family helix-turn-helix domain but lacked the LexA/signal peptidase superfamily domain common to other characterized phage repressors within this family (34). ΦD also harbored an ORF (SUFP_063) with homology to the single-stranded binding protein family whose members were involved in DNA replication via binding of ssDNA at the primosome assembly site. ΦA also possessed two ORFs that belong to the XRE-superfamily and lacked specific catalytic domains (SUFA_030 and SUFA_031). While these proteins could be mapped to broad protein families, it was not yet feasible to elucidate function based on these assignments.

Relevant to the present study was the observation that CB-A showed measurable prophage induction not evident in CB-D when these strains were grown in a complex medium. Prophage induction was readily assessed by quantifying the number of free phages in the growth medium and can influence host growth dynamics as a fraction of the population undergoes viral-mediated lysis (29). It was feasible that the prophage provides some fitness advantage to their host as neither is lost by routine passaging in the lab (unpublished data).

Here, we compared the metabolite and lipid profiles of each host-phage pair (CB-A and CB-D) grown on different carbon sources. The three mediums utilized represented a spectrum of the complexity of carbon substrates and nutrients, ranging from simple to complex and inorganic to organic, respectively (acetate < glutamate < tryptone + yeast extract) (Table S1 and Materials and Methods).

10.1128/msystems.00585-22.1 TABLE S1 Total carbon, organic nitrogen, inorganic nitrogen, combined nitrogen molar concentrations, and C:N ratios for all mediums used in this study. Download Table S1, DOCX file, 0.01 MB. Copyright © 2022 Basso et al. 2022 Basso et al. https://creativecommons.org/licenses/by/4.0/ This content is distributed under the terms of the Creative Commons Attribution 4.0 International license.

### Growth physiologies of strains were different across substrates.

**FIG 1:**
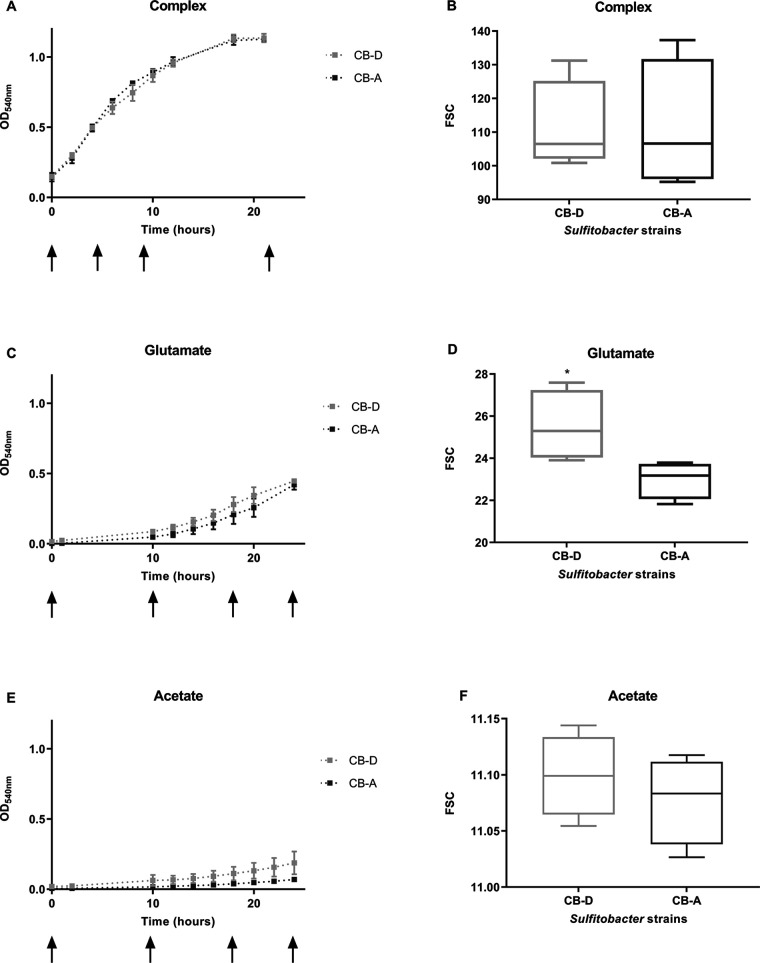
Growth dynamics and cell size data for strains CB-D and CB-A provided (A) a complex medium (with yeast extract and tryptone), (C) a defined medium (with glutamate as sole carbon source), and (E) a defined medium (with acetate as sole carbon source). Cell size was measured by forward scattering through flow cytometry for cells grown with (B) complex, (D) glutamate-containing, and (F) acetate-containing medium. Final time point average (with standard deviation) viable counts of each substrate per strain is as follows. Complex medium CB-A = 5.36 × 10^9^ (± 5.11 × 10^8^); CB-D = 4.37 × 10^9^ (± 4.9 × 10^8^). Glutamate medium: CB-A = 1.54 × 10^9^ (± 4.90 × 10^8^); CB-D = 2.02 × 10^9^ (± 7.16 × 10^8^). Acetate medium: CB-A = 2.85 × 10^8^ (± 1.07 × 10^7^); CB-D = 1.02 × 10^9^ (± 6.85 × 10^8^). Significant differences in cell size (Student’s *t* tests) are denoted by asterisks (*, *P* < 0.05). Averages of biological replicates are reported for all treatments (*n* = 3 for complex medium grown cells; *n* = 5 in glutamate and acetate grown cells). Plating was done in technical replicates. Arrows denote time points at which samples were taken from each medium type.

The growth of both strains was most robust on the complex substrate, followed by glutamate then acetate (Fig. 1A, C, and E). The strains used in these studies likely preferred the organic N present in both the complex and glutamate medium over the inorganic form provided in the base medium (20). Thus, the absence of an exogenous source of organic N in the acetate-based medium may have contributed to the strains’ poor growth on this substrate. As expected, when the strains were grown on the defined medium (containing either glutamate or acetate) the cells were smaller relative to complex medium-grown cells (Fig. 1B, D, and F; (35)). Free phages were present in all CB-A cultures but undetectable in the CB-D cultures (Table S2). Only the acetate-grown cultures showed significant differences in between-strain growth dynamics, presumably because of greater prophage induction in CB-A (Table S2). By the final (24 h) time point, viable counts for CB-D were ~250% greater than CB-A in acetate-grown cultures relative to the other treatments (Table S3). Cell size, estimated by forward side scatter, revealed significantly larger CB-D cells relative to CB-A cells for glutamate-grown cells (Fig. 1B). No differences were evident between the strains reared on the other two substrates (Fig. 1D and F).

10.1128/msystems.00585-22.2 TABLE S2 Incidence of SPI for strains CB-D (blue) and CB-A (orange) cells grown in (A) complex, (B) glutamate, and (C) acetate media. Download Table S2, DOCX file, 0.1 MB. Copyright © 2022 Basso et al. 2022 Basso et al. https://creativecommons.org/licenses/by/4.0/ This content is distributed under the terms of the Creative Commons Attribution 4.0 International license.

10.1128/msystems.00585-22.3 TABLE S3 Optical density and viable counts data for strains CB-A and CB-D. These tables are shown for all substrates for cells grown in (A) complex (SMM), (B) glutamate, or (C) acetate mediums. Download Table S3, DOCX file, 0.05 MB. Copyright © 2022 Basso et al. 2022 Basso et al. https://creativecommons.org/licenses/by/4.0/ This content is distributed under the terms of the Creative Commons Attribution 4.0 International license.

### The diversity of detected metabolites varied strongly with the growth substrate.

**FIG 2:**
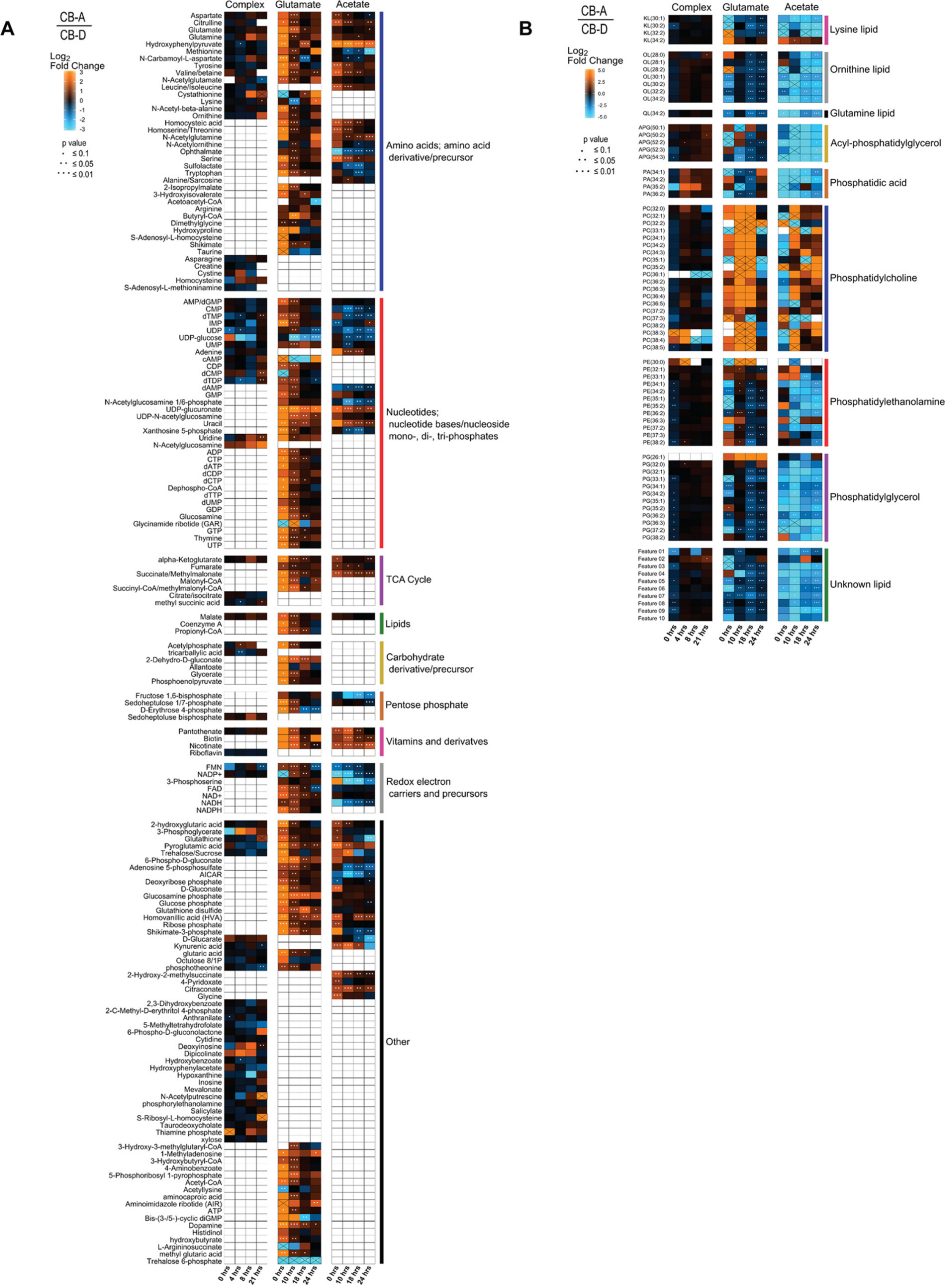
Metabolomics (A) and lipidomics (B) analysis of complex, glutamate, and acetate grown cells. Heat maps display fold differences between strains CB-D and CB-A over time. Significant differences are denoted by asterisks (*, *P* < 0.1; **, *P* < 0.05; ***, *P* < 0.01). Crossed boxes denote metabolites or lipids detected in only one strain, indicated by the color. Averages of biological replicates are reported for all treatments (*n* = 3 for complex medium grown cells; *n* = 5 in glutamate and acetate grown cells).

**FIG 3:**
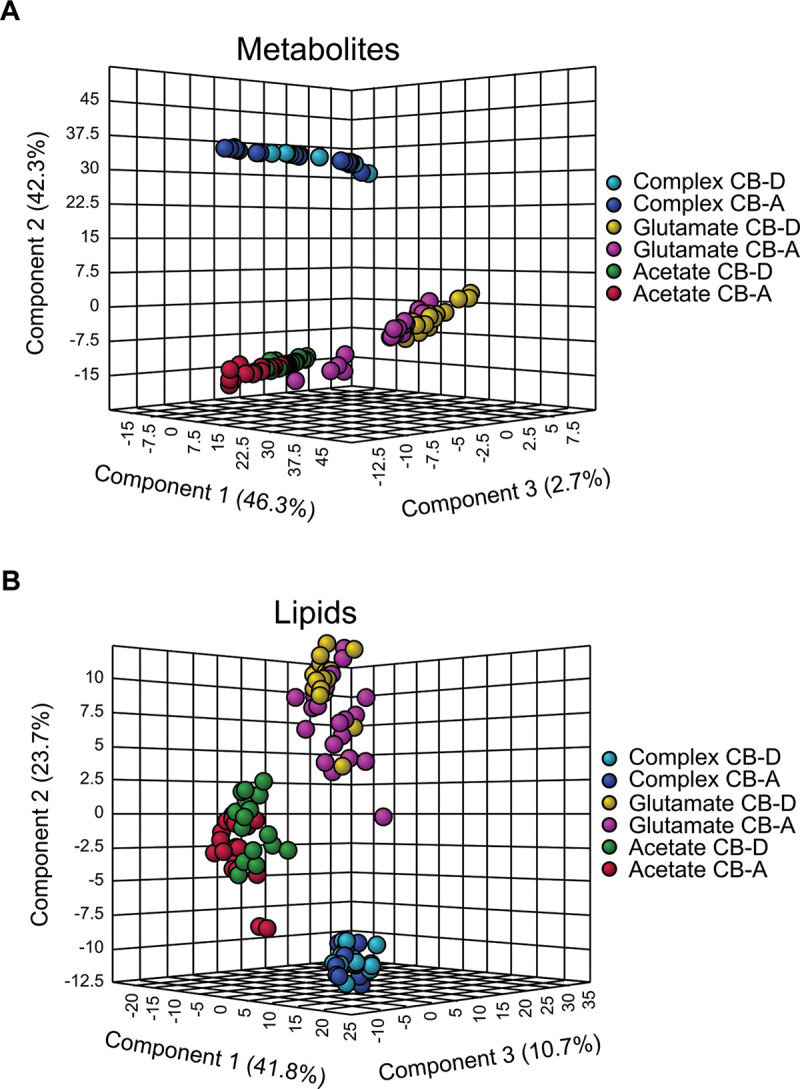
Partial least-squares discriminant analysis (PLS-DA) of strains CB-D and CB-A metabolites (A) and lipids (B). The following color scheme was used to denote samples: pink circles, acetate grown CB-A cells; green circles, acetate grown CB-D cells; dark blue circles, complex medium grown CB-A cells; light blue circles, complex medium grown CB-D grown cells; purple circles, glutamate grown CB-A cells; yellow circles, glutamate grown CB-D cells. All biological replicates are reported for all treatments (*n* = 3 for complex medium grown cells; *n* = 5 in glutamate and acetate grown cells). APG, acyl-phosphatidylglycerol.

All cultures were sampled for metabolomics and lipidomics analyses at four discrete time points throughout the growth curve. Using ultra-high-performance liquid chromatography-high resolution mass spectrometry (UHPLC-HRMS) based on untargeted metabolomics, a total of 175 central carbon metabolites were identified across all samples, with only 27 (15%) detected in all growth conditions (Fig. 2A). The greatest number of metabolites were detected in glutamate-grown cells (127 of the identified metabolites; 73%). Complex-grown cells and acetate-grown cells had similar numbers of detectable metabolites (75 and 72 identified metabolites, respectively; ~40%). There was some overlap of individual metabolites that were detected in cultures grown on these two substrates (31 metabolites), but more overlap was evident with glutamate-grown cells and either complex-or acetate-grown cells (40 and 63 metabolites, respectively). Partial least-squares discriminant analysis (PLS-DA) showed strong separation of samples by growth substrate, with little to no difference between strains (Fig. 3). Where evident, between-strain variation was greatest at discrete time points. For example, for both glutamate- and acetate-grown cultures, the greatest number of significant differences (fold difference >1.5 and *P* < 0.05) was observed at 10 h postinoculation (57% of metabolites for glutamate, 47% of metabolites for acetate). These between-strain differences in metabolite profiles diminished by the final sampling time point, but still comprised a third of measurable metabolites in the acetate-grown cultures (8% and 31% for glutamate- and acetate-grown cells) (Fig. S1A). The greatest difference in metabolite profiles for complex-grown cultures was 5% at 21 h.

10.1128/msystems.00585-22.4 FIG S1 Percentages of significantly different (A) metabolites and (B) lipids of complex, glutamate, and acetate grown cells. The significant difference is defined as fold change >1.5 and *P* < 0.05. Corresponding metabolomic and lipidomic heatmap analyses are shown in Fig. 2. Averages of biological replicates are reported for all treatments (*n* = 3 for complex grown cells; *n* = 5 for glutamate and acetate grown cells). Download FIG S1, PDF file, 0.5 MB. Copyright © 2022 Basso et al. 2022 Basso et al. https://creativecommons.org/licenses/by/4.0/ This content is distributed under the terms of the Creative Commons Attribution 4.0 International license.

### Lipid profiles varied with growth substrate and strain.

A total of 75 lipids from nine lipid classes were identified across all samples via UHPLC-HRMS-based untargeted lipidomics. Five phospholipid classes (phosphatidic acid [PA], phosphatidylethanolamine [PE], phosphatidylcholine [PC], phosphatidylglycerol [PG], and acyl-phosphatidylglycerol [APG]), three aminolipid classes (AL; lysine lipid [KL], ornithine lipid [OL], and glutamine lipid [QL]) and one class of Unknown lipid-like features (Unk) were detected. The lipid classes identified in this study were the same major (PC, PG, PE) and minor (APG, AL) classes found in other characterized *Sulfitobacter* species reared in a complex medium (Marine Broth 2216) (36). The presence of an unidentified lipid class in our strains is consistent with findings in other sulfitobacters (36). However, the methods of lipid identification employed (TLC versus UHPLC-HRMS) limit direct comparisons. A common bacterial phospholipid class, cardiolipin, has been reported as a minor lipid in roseobacters, including *Sulfitobacter* species (37). This lipid was not detected in our strains, likely because it is either not found in these strains or is present at levels below the limit of detection of the assay.

**FIG 4:**
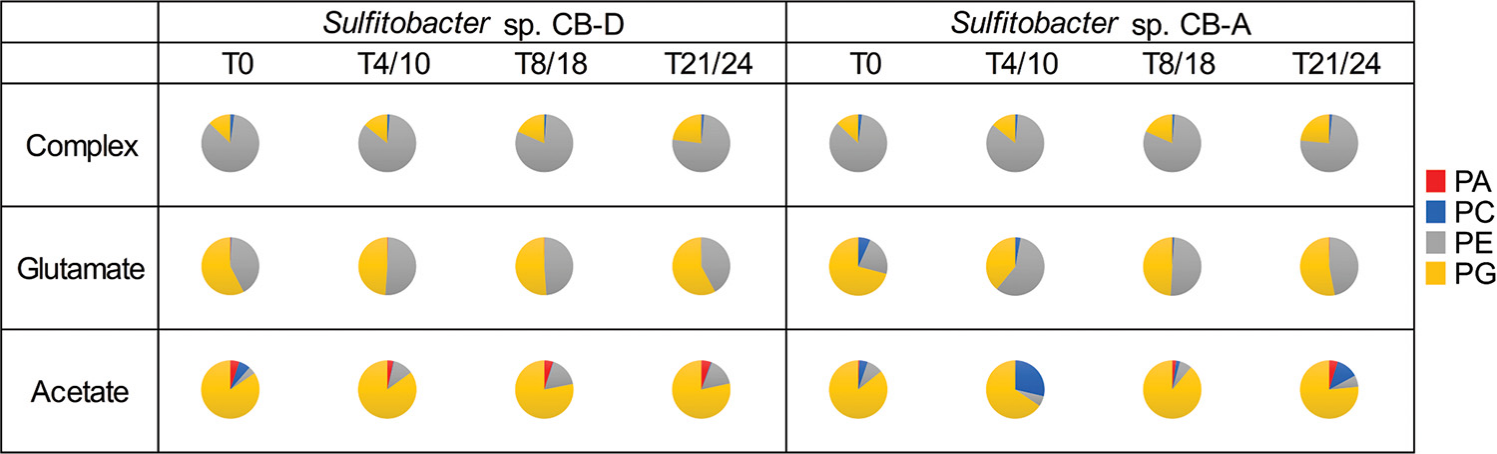
Pie charts display relative proportions of the major phospholipid concentrations in strains CB-D and CB-A for each time point and substrate. Temporal samplings were at 0, 4, 8, and 21 h for complex medium-grown cells and 0, 10, 18, and 24 h for defined medium with either glutamate or acetate as the sole carbon source. Phospholipid concentrations were determined using external calibration curves for phosphatidic acid (PA), phosphatidylcholine (PC), phosphatidylethanolamine (PE), and phosphatidylglycerol (PG). Averages of biological replicates are reported for all treatments (*n* = 3 for complex medium grown cells; *n* = 5 in glutamate and acetate grown cells) and were within 2 standard deviations. Pie charts displaying relative ion counts for all detected lipid classes are included in Fig. S3.

Unlike the metabolite profiles, most of these lipids (94 to 97%) were detected in all samples, regardless of growth medium or strain type (Fig. 2B). Thus, the differences between these profiles were principally due to alterations in the relative abundance of specific lipids. While the samples were principally clustered by growth substrate (Fig. 3), greater variation between strains raised on either glutamate or acetate was evident among these lipid profiles. For example, upwards of 45% of lipids showed significantly different distributions between the two strains grown on acetate (at 24 h), and 63% of lipids varied between the strains when grown on glutamate (at 18 h). In contrast, ≤8% of lipids were significantly different between the strains when grown on a complex medium (all time points) (Fig. S1B). Regardless of the growth substrate, significant changes were observed within the phospholipid profiles over time. When grown on complex media, the ratio of PG to the total phospholipids detected generally increased with time for both strains, while the ratio of PE to total phospholipids decreased. However, acetate-grown cells demonstrated the opposite trend. Glutamate-grown cells showed the ratio of PG increasing over time for CB-D, however, a consistent trend was not observed for CB-A. (Fig. 4). In addition to the obvious variation between growth substrates and temporal changes, a subset of lipid classes that showed significant variation between the two strains were the aminolipids.

10.1128/msystems.00585-22.6 FIG S3 Relative proportions of lipid intensities to total detected lipid intensities in strains CB-D and CB-A for each timepoint and substrate. Ratios are shown for phosphatidic acid (PA), phosphatidylcholine (PC), phosphatidylethanolamine (PE), phosphatidylglycerol (PG), lysine lipid (KL), ornithine lipid (OL), glutamine lipid (QL), and an unidentified class of lipid-like features. Averages of biological replicates are reported for all treatments (*n* = 3 for complex grown cells; *n* = 5 for glutamate and acetate grown cells) and are within 2 standard deviations. Download FIG S3, PDF file, 0.7 MB. Copyright © 2022 Basso et al. 2022 Basso et al. https://creativecommons.org/licenses/by/4.0/ This content is distributed under the terms of the Creative Commons Attribution 4.0 International license.

### Presence of aminolipids correlated with carbon substrate.

**FIG 5:**
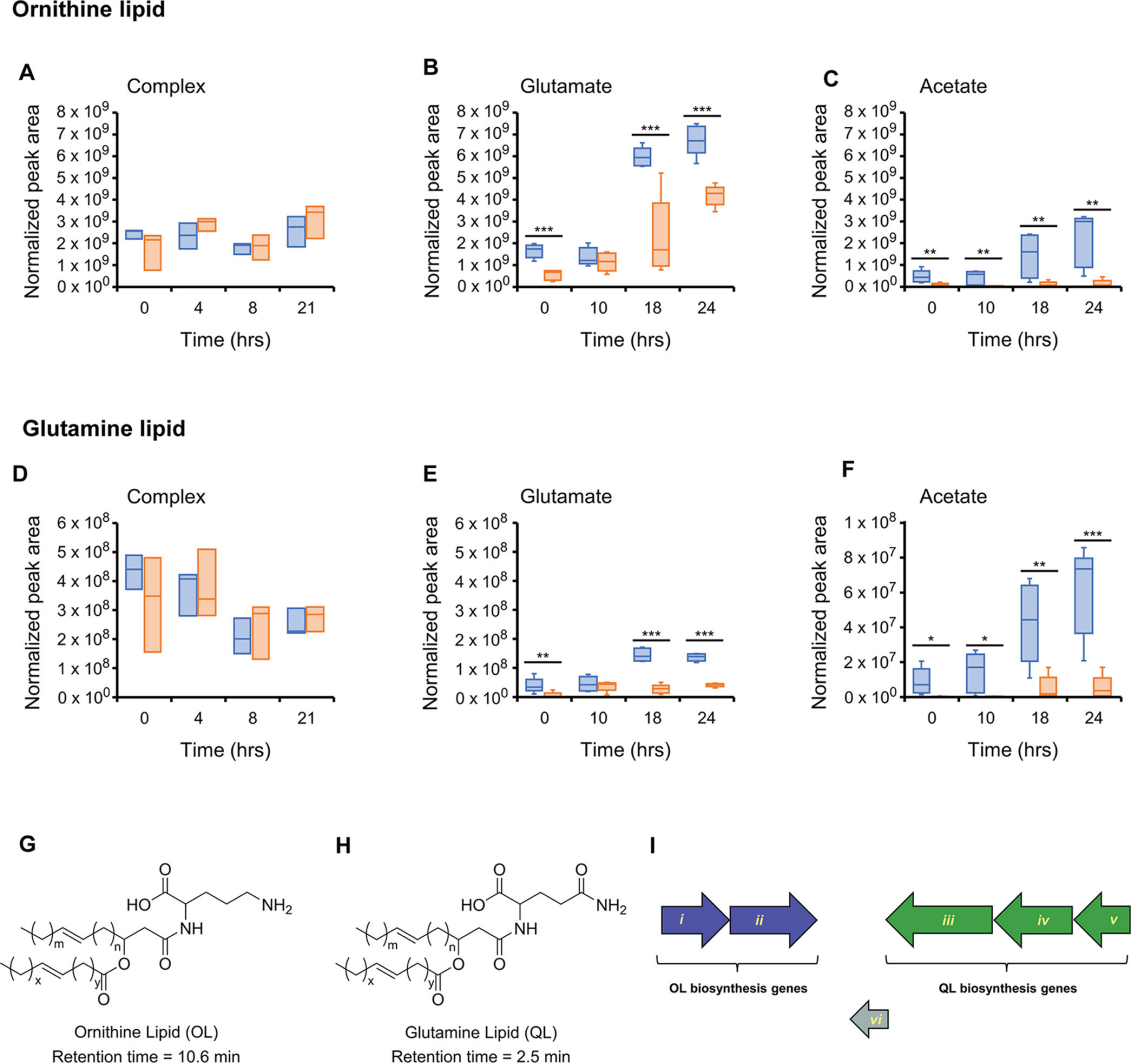
Aminolipids in strains CB-A and CB-D. Ornithine lipid (OL) and glutamate lipid (QL) for CB-D (blue) and CB-A (orange) grown on complex (A and D), glutamate (B and E), or acetate (C and F) mediums. Significant differences are denoted by asterisks (*, *P* < 0.1; **, *P* < 0.05; ***, *P* < 0.01). Averages of biological replicates are reported for all treatments (*n* = 3 for complex medium-grown cells; *n* = 5 in glutamate and acetate-grown cells). Structures of ornithine lipid (OL) (G) and glutamate lipid (QL) (H), with respective retention times. OL and QL biosynthesis gene organization in strains CB-D and CB-A. NCBI accession numbers are provided in parentheses. Strain CB-D (CP072613) has 3672 ORFs, while CB-A (PYUG00000000) has 3459 ORFs. (I) *i*, GNAT family N-acetyltransferase (WP 037944303.1), *ii, *1-acyl-sn-glycerol-3-phosphate acyltransferase (WP 037944304.1); *iii*, ATP-binding cassette domain-containing protein (WP 037944390.1); *iv*, ornithine-acyl-ACP acyltransferase (PTA99994); v, outer membrane protein assembly factor BamE (WP 037944392).

Ornithine (OL) and glutamine lipids (QL) have been previously characterized in Rhodobacter sphaeroides, an organism belonging to the same Rhodobacteraceae family as our host strain (38). Here, the identification of AL, determined through exact mass and proposed structures, was confirmed using all ion fragmentation (Fig. 5G and H) (39). The MS-based identification of these lipids is supported by genome analyses that indicated both strains possess homologs of genes previously shown to be essential for OL and QL biosynthesis in Silicibacter pomeroyi DSS-3, a close relative of sulfitobacters (Fig. 5I).

Of these lipids, a single glutamine lipid (QL 34:2), seven ornithine lipids (OL), and four lysine lipids (KL) were detected, with significant differences between strains when grown on glutamate or acetate (Fig. 5 and Fig. S2). Complex medium-grown cells showed relatively constant OL abundance over time (Fig. 5A). In contrast, in glutamate- and acetate-grown cultures, OL generally increased over time (Fig. 5B and C). In complex medium-grown cells, QL (34:2) was detected with decreasing abundance over time (Fig. 5D), but with increasing abundance over time in glutamate- and acetate-grown cells (Fig. 5E and F). Additionally, OL and QL were detected with significantly higher abundance for CB-D than CB-A in glutamate-grown cells at 0, 18, and 24 hand acetate-grown cells (all time points) (Fig. 5B, C, E, and F). In contrast, no KL was detected in glutamate-grown CB-A cells (Fig. S2) but followed a similar trend as OL and QL in acetate-grown cells with significantly higher abundance for CB-D than CB-A at 10, 18, and 24 h. In complex medium-grown cells, no difference in KL was evident between the strains, and abundance remained more constant until 21 h when an increase was observed.

10.1128/msystems.00585-22.5 FIG S2 Lysine lipid (KL) in strains CB-A and CB-D grown on (A) complex (B) glutamate, or (C) acetate-containing media. Significant differences are denoted by asterisks (*, *P* < 0.1; **, *P* < 0.05). Averages of biological replicates are reported for all treatments (*n* = 3 for complex grown cells; *n* = 5 for glutamate and acetate grown cells). Structures of (D) lysine lipid (KL), with respective retention time. Download FIG S2, PDF file, 0.6 MB. Copyright © 2022 Basso et al. 2022 Basso et al. https://creativecommons.org/licenses/by/4.0/ This content is distributed under the terms of the Creative Commons Attribution 4.0 International license.

### Detection of an abundant but unidentified lipid class.

In addition to the aforementioned phospholipids and aminolipids, a class of unidentified spectral features with characteristics of lipids (subsequently termed “unidentified lipid class”) was detected in both CB-D and CB-A. This unidentified lipid class was highly abundant in glutamate- and acetate-grown cells. This lipid class was most abundant at the first sampling time point (0 h; immediately the following subculture), with the ratio of this lipid class to the total detected lipids reaching values of ~35% in glutamate-grown cultures of both strains and 52% and 56% in acetate-grown CB-D and CB-A cultures, respectively (Fig. S3). This lipid class remained highly abundant with the lowest ratios reaching values of ~20% for CB-D and CB-A grown on glutamate (18 h), and 40% for CB-D (24 h) and 33% for CB-A (10 h) when grown on acetate (Fig. S3). However, this lipid class was not as abundant in the complex medium-grown cultures of either strain, with ratios of only 3% at 0 h, and a maximum ratio of ~13% at 21 h (Fig. S3). This unknown lipid class displayed significant differences between strains grown on either of the defined carbon substrate conditions, but not on the complex medium (Fig. 2B), with the most significant differences in glutamate-grown cells. Between-strain differences in glutamate were ≥60% at all time points, except 10 h at which only 10% of features were significantly different (Fig. 2B). Acetate-grown cells showed less deviation throughout the growth curve, but had dramatic differences (70% of this class of features) at 24 h (Fig. 2B).

To gain a better understanding of these features, relative abundances of mass spectral isotope peaks were compared to the natural abundance of carbon-13 (1.1%). The mass-to-charge (*m/z*) ratios for these features ranged from 702.4363 to 816.5776 *m/z* (±5 ppm), with the most abundant species at 802.5621 *m/z*, all in negative ionization mode for the [M-H]-ion. The corresponding masses for the [M+H]^+^ ion were detected using positive ionization mode. From these observed masses, using the nitrogen rule, it was suggested that the lipid-like features contained an odd number of nitrogen atoms. To gain further understanding of this class of features, further fragmentation experiments were performed using a Q Exactive quadrupole-orbitrap hybrid mass spectrometer. Using Fiehn’s seven golden rules (40), 137 potential molecular formulae were determined for the most abundant feature (802.5621 *m/z*), containing carbon, hydrogen, nitrogen, oxygen, phosphorus, or sulfur. These features were compared with online lipid databases, including LipidMaps (41), but currently elude identification.

## DISCUSSION

The ability of bacteria to modulate the lipid composition of their cellular membranes is a common adaptive mechanism to environmental fluctuations. From a biogeochemical standpoint, much of the existing knowledge regarding factors influencing lipid composition in marine bacteria focuses on the role of phosphorus (P) availability, revealing the widespread capacity for marine microbes to remodel their membrane lipids in response to starvation of this element (e.g., (42, 43)). However, lipid biosynthesis is intrinsically linked to, and dependent on, central metabolism. The latter of which operates on a shorter timescale than the former (44, 45). As such, the role of primary growth substrates can be predicted to be a critical factor in shaping cellular lipid composition. Here, we sought to obtain a more holistic picture of the relationship between growth regimes, metabolism, and lipid composition in a representative heterotrophic marine bacterium of the *Roseobacter* clade of marine bacteria. A secondary goal was to assess the contribution of phage activity to the host cell metabolome and lipidome. To this end, we compared two genetically similar host-phage pairs, with different intrinsic levels of spontaneous induction (i.e., CB-A cultures produce measurable free phage particles in the absence of obvious stressors, CB-D does not; 33). Disentangling responses due to growth conditions, host physiology, and viral activity is difficult due to interdependencies. Nonetheless, the data presented here reveal stark differences in metabolite and lipid profiles which appear to be driven principally by growth regime and secondarily by prophage type.

*Sulfitobacter* metabolite profiles were better defined by growth conditions than strain-prophage pair. Across the three mediums, there was little (15%) overlap in the metabolites that were detected and identified. This is comparable to a study in Pseudomonas aeruginosa, in which only 40% of 145 detectable metabolites were found across all strains raised on six distinct carbon substrates (46). The strains grown on glutamate had the greatest number of detectable metabolites, 69% more than the strains grown on complex or acetate-containing media. Glutamate represents a major metabolite hub in many organisms. Beyond its involvement in protein synthesis, glutamate plays a central role in nitrogen assimilation, nucleoside, amino, and cofactor biosynthesis as well as the production of secondary metabolites. Furthermore, it has been reported as the most abundant of the water-soluble small metabolites in bacteria (20, 47). The high diversity of quantifiable metabolites in strains grown on this compound may reflect the diversity of metabolite pathways that this organism employs when given this compound as a primary growth substrate. A high concentration of glutamate may enable cells to more readily connect diverse pathways and direct this central metabolite into numerous anabolic and catabolic processes.

Metabolome differences between *Sulfitobacter* strains raised under the same growth conditions were evident and most pronounced in glutamate- and acetate-grown cultures. The overwhelming majority of these differences were the result of an increase in a given metabolite in the CB-A cells relative to CB-D. This finding is consistent with earlier studies in the same *Sulfitobacter* host strain that revealed a generalized increase in host metabolic activity (and in turn most measurable metabolites) in response to an obligately lytic viral infection. The increased metabolic demand in these cells was met by the recycling of metabolites released into the environment by lysed siblings (20). A similar scenario is likely in those CB-A cultures with spontaneous induction leading to some cell lysis and resulting in a more diverse pool of carbon and nutrient sources available to the majority of surviving cells. While this could be expected to contribute to the observed between-strain metabolite and lipid differences, it is worth noting that no significant growth differences were observed between the two strains when grown on glutamate, strongly suggesting that only a small fraction of cells were undergoing lysis. Obtaining more definitive information on the proportion of the population that succumbs to lysis during induction on the different mediums would help elucidate the contribution of lysed cell constituents to the observed metabolite and lipid profiles.

The lipid profiles support the metabolite data in that strains can be discriminated by growth medium and strain-phage pairs. Unlike the metabolite profiles, nearly all detected lipids were present across all samples. Mirroring the metabolite profiling, strain level differences were not strongly apparent in complex medium-grown cultures, but significant between strains grown on either of the defined media, resulting in upwards of 50% and 60% variation in lipid profiles between the strains when grown on acetate and glutamate, respectively. The observed variation was noted in both the overall composition of the lipid classes as well as in the composition of the acyl moieties. Temporal variation in lipid profiles for batch-grown cultures is expected and anticipated to be the result of changing environmental conditions that occur in a closed system (e.g., changes in nutrient concentrations, accumulation of metabolite waste products, alterations in oxygen levels) (9). As such, we caution against drawing summative conclusions regarding the temporal variation evident in these lipid profiles but do note the observed variation demonstrates flexibility in lipid composition in these strains that should be taken into consideration in future experiments to characterize lipid composition in these and related strains.

The most striking difference in lipid composition across medium types occurred within two major phospholipid classes: PE and PG. These two lipid classes typically co-occurred in diverse bacteria. Molecular simulations suggested interactions between the two influenced membrane integrity; increases in PG relative to PE are predicted to increase membrane stability and decrease membrane permeability (48). We observed that PE was the dominant phospholipid in complex medium-grown cells whereas PG dominated in the glutamate- and acetate-grown cells, suggestive of a fundamentally different membrane architecture when these strains were grown on defined versus complex media. Given common parameters influencing lipid composition in bacteria were held static across the medium types (i.e., pH, salinity, and temperature), we interpreted the differences were due to other components of the medium, namely, the primary growth substrate(s) and/or nutrients. This contrasted with studies in the nonmarine bacterium, E. coli, which indicated no significant difference in phospholipid composition in strains grown on a complex versus minimal medium (49). However, it has been noted that the membrane composition in E. coli is simple in comparison to many other bacteria species (9), which highlights the danger of using any single bacterium as a model for lipid membrane composition. These observations also corroborate previous work indicating that bacterial membranes are diverse in composition and that alterations in lipid profiles are a necessary adaptive response to environmental alterations (9).

Between-strain differences in lipid profiles were most evident in the ornithine (OL) and glutamine (QL) lipid classes and strains were grown on glutamate and acetate. The general trend was an increased abundance of these ALs in CB-D relative to CB-A, the strain with detectable prophage induction. It has been proposed that OL may lead to enhanced membrane stability (reviewed in reference (50)), and this lipid is required for optimal c-type cytochrome function in the alpha-Proteobacterium Rhodobacter capsulatus, where it has a role in managing oxidative stress (51). In addition, OL is overrepresented in the lipids of planktonic cells relative to their biofilm-grown siblings in the marine bacterium Pseudoalteromonas lipolytica, suggesting a role for this class of lipids in growth modality (11). While our results indicated nutrient concentration and prophage induction were discrete effectors of lipid composition, they most likely also had a combined influence. It was unclear whether these effects were direct (i.e., via metabolic routing tied to nutrient sources), indirect (i.e., a general stress response to the profile of available nutrients and/or lytic infection) or a combination of the two. Nonetheless, given evidence that lipid composition can have broad-range effects on cellular physiology, including enhancing susceptibility to environmental stress (49), it is feasible that the lipid composition of CB-A results from positive feedback between distinct cellular stressors mediated by phospholipid-dependent and phage-mediated processes in this strain. Finally, in an unexpected finding, a highly significant, and abundant (upwards of 50% of the total lipids in CB-A cultures grown on acetate) lipid-like class of spectral features was detected. As with the aminolipids, these features displayed notable differences between strains raised on the same growth medium. As such, these features are of particular interest for future study.

The bacterial metabolome comprises only a small fraction of the total cellular dry weight (~3%), and this collection of molecules primarily exists to provide energy and building blocks for macromolecular biosynthesis (47). Bacterial metabolism is highly responsive to substrate and nutrient availability, but it is also known to be influenced by viral infection. However, these forces were not unidirectional: viral infection is sensitive and responsive to cellular metabolism (e.g., (52)) as it is often wholly dependent upon the host cell processes to produce viral progeny. Because both nutrient fluctuations and virus pressure are common features of most natural ecosystems, including the coastal oceans where roseobacters dominate, the physiologies of resident bacteria are anticipated to be shaped by these intersecting factors. The metabolic and lipid profiling presented here provided a first look at the complexity of these interactions and laid the foundation for future studies that relate cellular composition with function.

## MATERIALS AND METHODS

### Bacterial propagation in different growth conditions.

*Sulfitobacter* sp. strains CB-A and C-D (formerly Sulfitobacter sp. strain CB2047; 33) were inoculated and incubated overnight in 10 mL cultures at 25°C at 200 rpm. Strains were grown in Standard Marine Media (SMM, here referred to as complex) (4.1 M NaCl; 950 mM KCl; 700 mM CaCl_2_; 20 mM H_3_BO_3_; 2.1 mM MgSO_4_ [7H_2_O]; 2.0 M MgCl_2_; 1.0 M Tris [Tris-HCl and Tris-Base; pH 7.5]; 800 mM NaHCO_3_; 5.0 M NH_4_Cl; 150 mM K_2_HPO_4_. 1.125 g yeast extract, 2 g tryptone, Fe, vitamins and minerals (53)), or Roseobacter Marine Media (RMM) (4 M NaCl; 0.2 M KCl; 0.2MCaCl2; 1.0 M MgSO_4_ [7H_2_O]; 1.0 M Tris-HCl [pH 7.5]; 0.5 M NH_4_Cl; 50 mM K_2_HPO_4_, Fe, vitamins and minerals (53)) supplemented with either (4 mM l-glutamic acid [glutamate], or 10 mM sodium acetate [acetate]). The concentrations of acetate and glutamate contained comparable amounts of carbon. There was variation in the nitrogen (N) and phosphorus (P) provided in the media. Inorganic N, as ammonium chloride, was supplied in all media. Organic N was available in both the complex and glutamate-containing media. Organic carbon in the complex medium was 8.55 times greater (171 mM) than either the glutamate or acetate-containing medium (both supplied at 20 mM). Combining organic and inorganic N sources, the complex medium (at 27 mM N) had 1.6 or 2.2 times greater total N than either the glutamate (14 mM) or acetate (10 mM) media, respectively. The C:N ratio for the three mediums ranged from 1.4 to 6.3 (glutamate to complex) (Table S1). In our experiments, P was provided in nonlimiting concentrations. Inorganic P was provided as 1 mM potassium phosphate, a nonlimiting concentration for roseobacters (39), in all media. Organic P was only available in the complex medium, as a component of both tryptone and yeast extract.

Overnight cultures (10 mL) of strains preconditioned on the medium type were subcultured into 200 mL volumes of fresh medium in 500 mL flasks (OD_540_ ≈ 0.17). Aliquots were immediately taken and indicated as “0 h” samples. Growth was monitored by OD_540_ every 2 h for 24 h. We note that the growth dynamics of the strains in large volume flasks differed from prior reports (29) in 10 mL test tubes. However, the induction phenotype remains consistent (i.e., CB-A produced measurable infectious particles in noninducing conditions while CB-D did not).

### Sampling methodology.

Aliquots were taken for metabolite and lipid analysis, as well as viable counts and spontaneous prophage induction, were taken at 0, 4, 8, and 21 h for complex grown cells and 0, 10, 18, and 24 h for glutamate and acetate grown cells as follows. For metabolite analysis, 5 mL of bacterial cells were rapidly collected (<30 sec) on Magna nylon filters (Millipore) via vacuum filtration and immediately placed in extraction solvent to quench metabolism (see subsequent section). For lipid analysis, 5 mL of bacterial cells were centrifuged at 4,000 rpm for 5 min at 4°C. This process pelleted cells but not free viruses. Lipids were extracted from pelleted cells within an hour of collection. For phage enumeration, 1 mL of bacterial culture was filtered through a 0.22 μm syringe filter and the cell-free medium was assayed as described below. For viable counts, 1 mL of bacterial culture was taken for serial dilutions, plating 10^−5^, 10^−6^, and 10^−7^ dilutions onto complex medium agar plates, in technical triplicate.

### Metabolite data collection and analysis.

Water-soluble metabolites were extracted from filtered samples using 4:4:2 acetonitrile:methanol:water with 0.1 M formic acid as previously described (54). Analysis of the extracted metabolites was carried out using UHPLC-HRMS (Thermo Scientific, San Jose, CA, USA) with a previously validated untargeted metabolomics method (55). The metabolites were separated using reversed-phase chromatography utilizing a Synergi Hydro RP column (100mmx 2.1 mm, 2.6 μm, 100 Å; Phenomenex, Torrance, CA) and an UltiMate 3000 pump (Thermo Scientific). All solvents used were HPLC grade. An Exactive Plus Orbitrap MS (Thermo Scientific) was used for the full scan mass analysis. For the glutamate- and acetate-grown cells, each biological replicate was analyzed by UHPLC-HRMS in triplicate. Following the mass analysis, metabolites were identified by exact mass and retention time from an in-house standard library using the open-source software package, Metabolomics Analysis and Visualization Engine (MAVEN) (56, 57). The area under the curve (AUC) was integrated and normalized to cell density (optical density at 450 nm [OD_540_]) to obtain per cell metabolite abundances. It was confirmed that the relationship between OD and viable cells is similar for the two strains within a given medium type. Ratios of these values from each strain at a given time point and growth condition were used to determine relative concentrations of metabolites.

### Lipid data collection and analysis.

Lipids were extracted from cell pellets using 15:15:5:1:0.18 95% ethanol, water, diethyl ether, pyridine, and 4.2 N ammonium hydroxide followed by water-saturated butanol extraction according to the protocol for glycerophospholipids and sphingolipids described previously (58). The extracted lipids were dried under a steady stream of nitrogen and resuspended in 300 μL of a 9:1 ratio of methanol:chloroform before UHPLC-HRMS analysis. Extracted lipids were analyzed by UHPLC-HRMS using an established untargeted lipidomics method (59). The chromatographic separations were performed using a Kinetex HILIC column (150 mm × 2.1 mm, 2.6 μm, 100 Å; Phenomenex) and an UltiMate 3000 pump (Thermo Scientific). The full scan mass analysis was carried out in both positive and negative ionization modes with an Exactive Plus Orbitrap MS (Thermo Scientific). Glutamate- and acetate-grown cells were analyzed in triplicate by UHPLC-HRMS. The phospholipids were then identified by exact mass and retention time by comparison to an in-house standard library using MAVEN. Aminolipids were also identified by exact mass using MAVEN. Aminolipids were confirmed using isotopic patterns as well as fragmentation data gathered by all ion fragmentation utilizing high energy collision dissociation (HCD). Aminolipids KL, OL, and QL were detected at retention times of 10.7, 10.6, and 2.5 min, respectively. Statistical analyses were performed on the OD normalized AUC as done for the metabolite analysis.

### Phage enumeration.

Filter-sterilized (cell-free) spent culture medium was quantified for infectious phage particles using a standard plaque assay (60). Briefly, 500 μL of susceptible host cultures (OD_540_ ≈ 0.17) was added to 3 mL top (0.55% to 0.60%) Noble agar aliquots and poured on agar base plates. Once the top agar dried, 10 μL of cell-free spent medium 10-fold serial dilutions were spotted in technical triplicate. The schematic included medium-only negative controls. Plates were incubated at room temperature, and zones of the clearing were observed 24 to 48 h after plating.

### Statistical analysis.

Heatmaps were prepared using the R statistical program (version 3.5.1). Fold differences were log_2_ transformed. Partial least-squares discriminant analysis (PLS-DA) was performed using MetaboAnalyst 4.0 (61). Before PLS-DA, data were filtered using interquartile range (IQR), log_2_ transformed, and Pareto scaled. PLS-DA was used to determine the relationship between two matrices to visualize differences between organism groups and substrates. The JGI Integrated Microbial Genomes (IMG) portal (img.jgi.doe.gov) was used to search for the OL and QL biosynthesis gene organization in the *Sulfitobacter* sp. strains CB-D (CP072613; 3672 ORFs) and CB-A genome (PYUG00000000; 3459 ORFs).

### Data availability.

The raw MS data are available at the MetaboLights database (https://www.ebi.ac.uk/metabolights/) under study identifier MTBLS5345.

## References

[1] Westfall CS, Levin PA. 2018. Comprehensive analysis of central carbon metabolism illuminates connections between nutrient availability, growth rate, and cell morphology in *Escherichia coli*. PLoS Genet 14:e1007205-25. doi:10.1371/journal.pgen.1007205. 29432413 PMC5825171

[2] Vadia S, Tse JL, Lucena R, Yang Z, Kellogg DR, Wang JD, Levin PA. 2017. Fatty acid availability sets cell envelope capacity and dictates microbial cell size. Curr Biol 27:1757–1767. doi:10.1016/j.cub.2017.05.076. 28602657 PMC5551417

[3] Catucci L, Depalo N, Lattanzio VMT, Agostiano A, Corcelli A. 2004. Neosynthesis of cardiolipin in *Rhodobacter sphaeroides* under osmotic stress. Biochemistry 43:15066–15072. doi:10.1021/bi048802k. 15554714

[4] Jackson M, Crick DC, Brennan PJ. 2000. Phosphatidylinositol is an essential phospholipid of mycobacteria. J Biol Chem 275:30092–30099. doi:10.1074/jbc.M004658200. 10889206

[5] Nguyen NA, Sallans L, Kaneshiro ES. 2008. The major glycerophospholipids of the predatory and parasitic bacterium *Bdellovibrio bacteriovorus* HID5. Lipids 43:1053–1063. doi:10.1007/s11745-008-3235-9. 18818966

[6] Park S, Jung Y-T, Won S-M, Park J-M, Yoon J-H. 2015. *Sulfitobacter undariae* sp. nov., isolated from a brown algae reservoir. Int J Syst Evol Microbiol 65:1672–1678. doi:10.1099/ijs.0.000156. 25724746

[7] Yao J, Rock CO. 2013. Phosphatidic acid synthesis in bacteria. Biochim Biophys Acta 1831:495–502. doi:10.1016/j.bbalip.2012.08.018. 22981714 PMC3548993

[8] Gao J-L, Weissenmayer B, Taylor AM, Thomas-Oates J, López-Lara IM, Geiger O. 2004. Identification of a gene required for the formation of lyso-ornithine lipid, an intermediate in the biosynthesis of ornithine-containing lipids. Mol Microbiol 53:1757–1770. doi:10.1111/j.1365-2958.2004.04240.x. 15341653

[9] Sohlenkamp C, Geiger O. 2016. Bacterial membrane lipids: diversity in structures and pathways. FEMS Microbiol Rev 40:133–159. doi:10.1093/femsre/fuv008. 25862689

[10] Weissenmayer B, Gao J-L, López-Lara IM, Geiger O. 2002. Identification of a gene required for the biosynthesis of ornithine-derived lipids. Mol Microbiol 45:721–733. doi:10.1046/j.1365-2958.2002.03043.x. 12139618

[11] Favre L, Ortalo-Magne A, Pichereaux C, Gargaros A, Burlet-Schiltz O, Cotelle V, Culioli G. 2018. Metabolome and proteome changes between biofilm and planktonic phenotypes of the marine bacterium *Pseudoalteromonas lipolytica* TC8. Biofouling 34:132–148. doi:10.1080/08927014.2017.1413551. 29319346

[12] Breitbart M, Bonnain C, Malki K, Sawaya NA. 2018. Phage puppet masters of the marine microbial realm. Nat Microbiol 3:754–766. doi:10.1038/s41564-018-0166-y. 29867096

[13] Lindell D, Sullivan MB, Johnson ZI, Tolonen AC, Rohwer F, Chisholm SW. 2004. Transfer of photosynthesis genes to and from *Prochlorococcus* viruses. Proc Natl Acad Sci USA 101:11013–11018. doi:10.1073/pnas.0401526101. 15256601 PMC503735

[14] Thompson LR, Zeng Q, Kelly L, Huang KH, Singer AU, Stubbe J, Chisholm SW. 2011. Phage auxiliary metabolic genes and the redirection of cyanobacterial host carbon metabolism. Proc Natl Acad Sci USA 108:E757–E764. doi:10.1073/pnas.1102164108. 21844365 PMC3182688

[15] Hurwitz BL, Hallam SJ, Sullivan MB. 2013. Metabolic reprogramming by viruses in the sunlit and dark ocean. Genome Biol 14:R123. doi:10.1186/gb-2013-14-11-r123. 24200126 PMC4053976

[16] Warwick-Dugdale J, Buchholz HH, Allen MJ, Temperton B. 2019. Host-hijacking and planktonic piracy: how phages command the microbial high seas. Virol J 16:13. doi:10.1186/s12985-019-1120-1. 30709355 PMC6359870

[17] Czyz A, Los M, Wrobel B, Wegrzyn G. 2001. Inhibition of spontaneous induction of lambdoid prophages in Escherichia colicultures: simple procedures with possible biotechnological applications. BMC Biotechnol 1:1–5. doi:10.1186/1472-6750-1-1. 11316465 PMC32160

[18] Howes WV. 1965. Effect of glucose on the capacity of *Escherichia coli* to be infected by a virulent λ bacteriophage. J Bacteriol 90:1188–1193. doi:10.1128/jb.90.5.1188-1193.1965. 5321475 PMC315801

[19] Roitman S, Hornung E, Flores-Uribe J, Sharon I, Feussner I, Beja O. 2018. Cyanophage-encoded lipid desaturases: oceanic distribution, diversity and function. ISME J 12:343–355. doi:10.1038/ismej.2017.159. 29028002 PMC5776448

[20] Ankrah NYD, May AL, Middleton JL, Jones DR, Hadden MK, Gooding JR, LeCleir GR, Wilhelm SW, Campagna SR, Buchan A. 2014. Phage infection of an environmentally relevant marine bacterium alters host metabolism and lysate composition. ISME J 8:1089–1100. doi:10.1038/ismej.2013.216. 24304672 PMC3996693

[21] De Smet J, Zimmermann M, Kogadeeva M, Ceyssens PJ, Vermaelen W, Blasdel B, Jang HB, Sauer U, Lavigne R. 2016. High coverage metabolomics analysis reveals phage-specific alterations to Pseudomonas aeruginosa physiology during infection. ISME J 10:1823–1835. doi:10.1038/ismej.2016.3. 26882266 PMC5029163

[22] Kido Soule MC, Longnecker K, Johnson WM, Kujawinski EB. 2015. Environmental metabolomics: analytical strategies. Marine Chemistry 177:374–387. doi:10.1016/j.marchem.2015.06.029.

[23] Boysen AK, Carlson LT, Durham BP, Groussman RD, Aylward FO, Ribalet F, Heal KR, White AE, DeLong EF, Armbrust EV, Ingalls AE, Bowman J. 2021. Particulate metabolites and transcripts reflect diel oscillations of microbial activity in the surface ocean. mSystems 6:e00896-20. doi:10.1128/mSystems.00896-20. 33947808 PMC8269247

[24] Withers E, Hill PW, Chadwick DR, Jones DL. 2020. Use of untargeted metabolomics for assessing soil quality and microbial function. Soil Biol Biochem 143:107758–107759. doi:10.1016/j.soilbio.2020.107758.

[25] Gaubert-Boussarie J, Prado S, Hubas C. 2020. An untargeted metabolomic approach for microphytobenthic biofilms in intertidal mudflats. Front Mar Sci 7:250. doi:10.3389/fmars.2020.00250.

[26] Buchan A, LeCleir GR, Gulvik CA, Gonzalez JM. 2014. Master recyclers: features and functions of bacteria associated with phytoplankton blooms. Nat Rev Microbiol 12:686–698. doi:10.1038/nrmicro3326. 25134618

[27] Touchon M, Bernheim A, Rocha EPC. 2016. Genetic and life-history traits associated with the distribution of prophages in bacteria. ISME J 10:2744–2754. doi:10.1038/ismej.2016.47. 27015004 PMC5113838

[28] Casjens S. 2003. Prophages and bacterial genomics: what have we learned so far? Mol Microbiol 49:277–300. doi:10.1046/j.1365-2958.2003.03580.x. 12886937

[29] Basso JTR, Ankrah NYD, Tuttle MJ, Grossman AS, Sandaa R-A, Buchan A. 2020. Genetically similar temperate phages form coalitions with their shared host that lead to niche-specific fitness effects. ISME J 14:1688–1700. doi:10.1038/s41396-020-0637-z. 32242083 PMC7305329

[30] Howard-Varona C, Hargreaves KR, Abedon ST, Sullivan MB. 2017. Lysogeny in nature: mechanisms, impact and ecology of temperate phages. ISME J 11:1511–1520. doi:10.1038/ismej.2017.16. 28291233 PMC5520141

[31] Ankrah NYD, Budinoff CR, Wilson WH, Wilhelm SW, Buchan A. 2014. Genome sequences of two temperate phages, ΦCB2047-A and ΦCB2047-C, infecting *Sulfitobacter* sp. strain 2047. Genome Announc 2:e00108-14. doi:10.1128/genomeA.00108-14. 24903862 PMC4047441

[32] Ankrah NYD, Lane T, Budinoff CR, Hadden MK, Buchan A. 2014. Draft genome sequence of *Sulfitobacter* sp. CB2047, a member of the *Roseobacter* clade of marine bacteria, isolated from an *Emiliania huxleyi* Bloom. Genome Announc 2:e01125-14. doi:10.1128/genomeA.01125-14. 25377705 PMC4223456

[33] Chaturongakul S, Ounjai P. 2014. Phage-host interplay: examples from tailed phages and Gram-negative bacterial pathogens. Front Microbiol 5:442. doi:10.3389/fmicb.2014.00442. 25191318 PMC4138488

[34] Sauer RT, Yocum RR, Doolittle RF, Lewis M, Pabo CO. 1982. Homology among DNA-Binding proteins suggests use of a conserved super-secondary structure. Nature 298:447–451. doi:10.1038/298447a0. 6896364

[35] Cesar S, Huang KC. 2017. Thinking big: the tunability of bacterial cell size. FEMS Microbiol Rev 41:672–678. doi:10.1093/femsre/fux026. 28961755 PMC5812501

[36] Zachariah S, Kumari P, Das SK. 2017. *Sulfitobacter pontiacus* subsp. *fungiae* subsp. nov., isolated from coral *Fungia seychellensis* from Andaman Sea, and description of *Sulfitobacter pontiacus* subsp. *pontiacus* subsp. nov. Curr Microbiol 74:404–412. doi:10.1007/s00284-017-1200-7. 28184991

[37] Pujalte MJ, Lucena T, Ruvira MA, Arahal DR, Macián MC. 2014. The Family Rhodobacteraceae, p 439–512. *In* Rosenberg E, DeLong EF, Lory S, Stackebrandt E, Thompson F (ed), The Prokaryotes: Alphaproteobacteria and Betaproteobacteria. Springer Berlin Heidelberg, Berlin, Heidelberg.

[38] Zhang X, Ferguson-Miller SM, Reid GE. 2009. Characterization of ornithine and glutamine lipids extracted from cell membranes of *Rhodobacter sphaeroides*. J Am Soc Mass Spectrom 20:198–212. doi:10.1016/j.jasms.2008.08.017. 18835523 PMC2779474

[39] Smith AF, Rihtman B, Stirrup R, Silvano E, Mausz MA, Scanlan DJ, Chen Y. 2019. Elucidation of glutamine lipid biosynthesis in marine bacteria reveals its importance under phosphorus deplete growth in Rhodobacteraceae. ISME J 13:39–49. doi:10.1038/s41396-018-0249-z. 30108306 PMC6298996

[40] Kind T, Fiehn O. 2007. Seven golden rules for heuristic filtering of molecular formulas obtained by accurate mass spectrometry. BMC Bioinformatics 8:1–20. doi:10.1186/1471-2105-8-105. 17389044 PMC1851972

[41] Fahy E, Subramaniam S, Murphy RC, Nishijima M, Raetz CR, Shimizu T, Spener F, van Meer G, Wakelam MJ, Dennis EA. 2009. Update of the LIPID MAPS comprehensive classification system for lipids. J Lipid Res 50 Suppl:S9–14. doi:10.1194/jlr.R800095-JLR200. 19098281 PMC2674711

[42] Carini P, Van Mooy BAS, Thrash JC, White A, Zhao Y, Campbell EO, Fredricks HF, Giovannoni SJ. 2015. SAR11 lipid renovation in response to phosphate starvation. Proc Natl Acad Sci USA 112:7767–7772. doi:10.1073/pnas.1505034112. 26056292 PMC4485111

[43] Van Mooy BA, Fredricks HF, Pedler BE, Dyhrman ST, Karl DM, Koblízek M, Lomas MW, Mincer TJ, Moore LR, Moutin T, Rappé MS, Webb EA. 2009. Phytoplankton in the ocean use non-phosphorus lipids in response to phosphorus scarcity. Nature 458:69–72. doi:10.1038/nature07659. 19182781

[44] Parsons JB, Rock CO. 2013. Bacterial lipids: metabolism and membrane homeostasis. Prog Lipid Res 52:249–276. doi:10.1016/j.plipres.2013.02.002. 23500459 PMC3665635

[45] Taymaz-Nikerel H, De Mey M, Baart G, Maertens J, Heijnen JJ, van Gulik W. 2013. Changes in substrate availability in *Escherichia coli* lead to rapid metabolite, flux and growth rate responses. Metab Eng 16:115–129. doi:10.1016/j.ymben.2013.01.004. 23370343

[46] Frimmersdorf E, Horatzek S, Pelnikevich A, Wiehlmann L, Schomburg D. 2010. How *Pseudomonas aeruginosa* adapts to various environments: a metabolomic approach. Environ Microbiol 12:1734–1747. doi:10.1111/j.1462-2920.2010.02253.x. 20553553

[47] Bennett BD, Kimball EH, Gao M, Osterhout R, Van Dien SJ, Rabinowitz JD. 2009. Absolute metabolite concentrations and implied enzyme active site occupancy in *Escherichia coli*. Nat Chem Biol 5:593–599. doi:10.1038/nchembio.186. 19561621 PMC2754216

[48] Zhao W, Róg T, Gurtovenko AA, Vattulainen I, Karttunen M. 2008. Role of phosphatidylglycerols in the stability of bacterial membranes. Biochimie 90:930–938. doi:10.1016/j.biochi.2008.02.025. 18373983

[49] Rowlett VW, Mallampalli VKPS, Karlstaedt A, Dowhan W, Taegtmeyer H, Margolin W, Vitrac H. 2017. Impact of membrane phospholipid alterations in *Escherichia coli* on cellular function and bacterial stress adaptation. J Bacteriol 199:e00849-16. doi:10.1128/JB.00849-16. 28439040 PMC5472821

[50] Vences-Guzman MA, Geiger O, Sohlenkamp C. 2012. Ornithine lipids and their structural modifications: from A to E and beyond. FEMS Microbiol Lett 335:1–10. doi:10.1111/j.1574-6968.2012.02623.x. 22724388

[51] Aygun-Sunar S, Mandaci S, Koch H-G, Murray IVJ, Goldfine H, Daldal F. 2006. Ornithine lipid is required for optimal steady-state amounts of c-type cytochromes in *Rhodobacter capsulatus*. Mol Microbiol 61:418–435. doi:10.1111/j.1365-2958.2006.05253.x. 16856942

[52] Clasen JL, Elser JJ. 2007. The effect of host *Chlorella* NC64A carbon: phosphorus ratio on the production of Paramecium bursaria *Chlorella* Virus-1. Freshwater Biology 52:112–122. doi:10.1111/j.1365-2427.2006.01677.x.

[53] Quigley LNM, Edwards A, Steen AD, Buchan A. 2019. Characterization of the interactive effects of labile and recalcitrant organic matter on microbial growth and metabolism. Frontiers in Marine Sciences 10:493. doi:10.3389/fmicb.2019.00493. PMC6433851 30941109

[54] Martin RM, Dearth SP, LeCleir GR, Campagna SR, Fozo EM, Zinser ER, Wilhelm SW. 2017. Microcystin-LR does not induce alterations to transcriptomic or metabolomic profiles of a model heterotrophic bacterium. PLoS One 12:e0189608-19. doi:10.1371/journal.pone.0189608. 29240841 PMC5730168

[55] Bazurto JV, Dearth SP, Tague ED, Campagna SR, Downs DM. 2017. Untargeted metabolomics confirms and extends the understanding of the impact of aminoimidazole carboxamide ribotide (AICAR) in the metabolic network of *Salmonella enterica*. Microb Cell 5:74–87. doi:10.15698/mic2018.02.613. 29417056 PMC5798407

[56] Clasquin MF, Melamud E, Rabinowitz JD. 2012. LC-MS data processing with MAVEN: a metabolomic analysis and visualization engine. Current Protocols in Bioinformatics 37:14.11.1–14.11.23. doi:10.1002/0471250953.bi1411s37. PMC4055029 22389014

[57] Melamud E, Vastag L, Rabinowitz JD. 2010. Metabolomic analysis and visualization engine for LC−MS Data. Anal Chem 82:9818–9826. doi:10.1021/ac1021166. 21049934 PMC5748896

[58] Guan XL, Riezman I, Wenk MR, Riezman H. 2010. Chapter 15 - Yeast lipid analysis and quantification by mass spectrometry, p 369–391, Methods in Enzymology, vol 470. Academic Press. 20946818 10.1016/S0076-6879(10)70015-X

[59] Tague ED, Woodall BM, Harp JR, Farmer AT, Fozo EM, Campagna SR. 2019. Expanding lipidomics coverage: effective ultra performance liquid chromatography-high resolution mass spectrometer methods for detection and quantitation of cardiolipin, phosphatidylglycerol, and lysyl-phosphatidylglycerol. Metabolomics 15:1–10. doi:10.1007/s11306-019-1512-7. PMC6947919 30919213

[60] Kropinski AM, Mazzocco A, Waddell TE, Lingohr E, Johnson RP. 2009. Enumeration of bacteriophages by double agar overlay plaque assay, p 69–76. *In* Clokie MRJ, Kropinski AM (ed), Bacteriophages: Methods and Protocols, Volume 1: Isolation, Characterization, and Interaction. Humana Press, Totowa, NJ. 10.1007/978-1-60327-164-6_7 19066811

[61] Chong J, Soufan O, Li C, Caraus I, Li S, Bourque G, Wishart DS, Xia J. 2018. MetaboAnalyst 4.0: towards more transparent and integrative metabolomics analysis. Nucleic Acids Res 46:W486–W494. doi:10.1093/nar/gky310. 29762782 PMC6030889

