# Supplementary material for: Growth Substrate and Prophage Induction Collectively Influence Metabolite and Lipid Profiles in a Marine Bacterium
Source: mSystems. 2022 Aug 16;7(5):e00585-22. doi: 10.1128/msystems.00585-22 (PMC9600351; doi:10.1128/msystems.00585-22)
Supplement: TABLE S1 [file msystems.00585-22-s0004.docx]

**Table S1.** Total carbon, organic nitrogen, inorganic nitrogen, combined nitrogen molar concentrations and C:N ratios for all media used in this study. The values for the complex medium are averages.

| **Totals (mM):** | **Complex** | **Glutamate** | **Acetate** |
| --- | --- | --- | --- |
| Carbon | 171.72 | 20.00 | 20.00 |
| Organic N | 22.31 | 4.00 | 0.00 |
| Inorganic N | 5.00 | 10.00 | 10.00 |
| Combined N | 27.31 | 14.00 | 10.00 |
|  |  |  |  |
| **Ratios:** | **Complex** | **Glutamate** | **Acetate** |
| C:N | 6.29 | 1.43 | 2.00 |
| C:N (organic N) | 7.70 | 5.00 | 0.00 |
| C:N (inorganic N) | 34.34 | 2.00 | 2.00 |
