# Supplementary material for: Growth Substrate and Prophage Induction Collectively Influence Metabolite and Lipid Profiles in a Marine Bacterium
Source: mSystems. 2022 Aug 16;7(5):e00585-22. doi: 10.1128/msystems.00585-22 (PMC9600351; doi:10.1128/msystems.00585-22)
Supplement: TABLE S2 [file msystems.00585-22-s0005.docx]

**Table S2.** Incidence of spontaneous prophage induction (SPI) for strains CB-D and CB-A cells grown in **(A)** complex, **(B)** glutamate, and **(C)** acetate media. Each prophage provides homoimmunity to superinfection, that is infection with the same virus genotype. Thus, the lysogen of the alternate phage genotype is used a suspectible host. SPI were estimated using spot plating assays using serial dilutions ranging from 10^-2^ to 10^-5^. Data for biological replicates are reported for all treatments and time points (n= 3 for complex grown cells; n = 5 for glutamate and acetate grown cells). Plating was done in technical triplicate. Detection of SPI is denoted by asterisks (* = detection at 10^-2^ dilution; ** = detection at 10^-3^ dilution; *** = detection at 10^-4^ dilution; **** = detection at 10^-5^ dilution; n.d. = not detected).

**
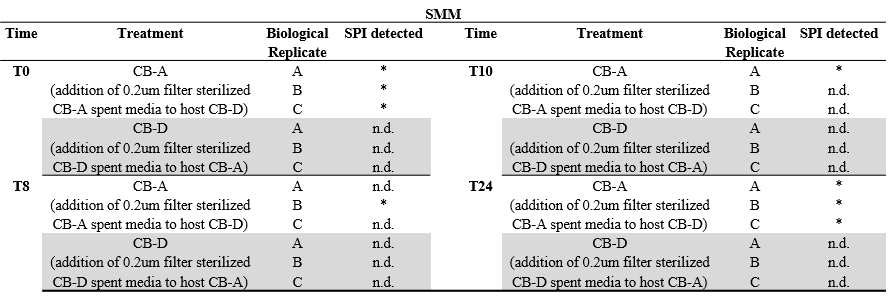

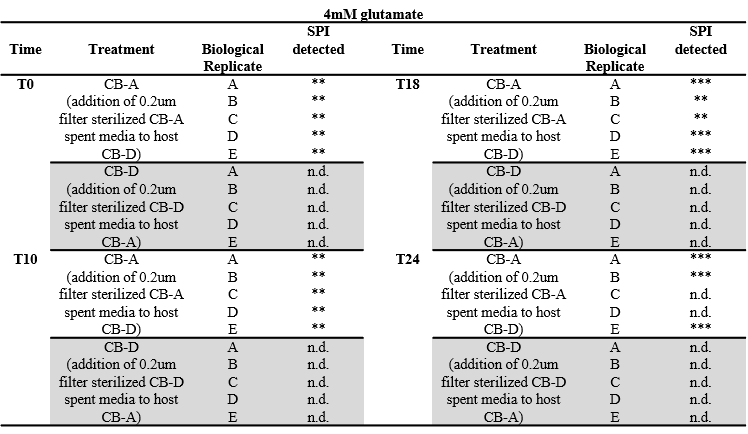

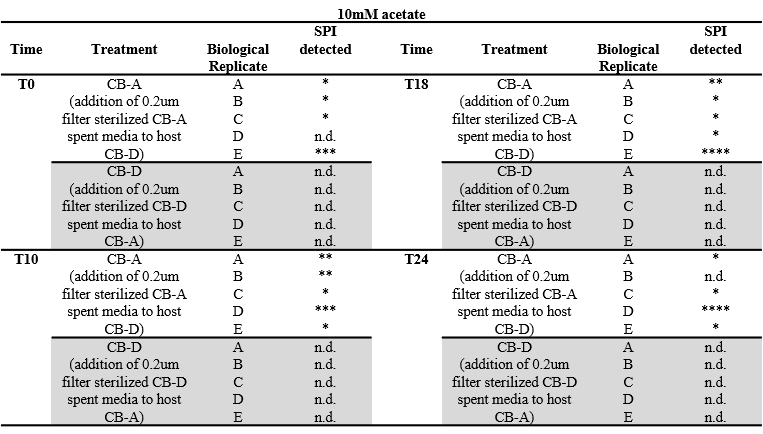
**

**A**

**B**

**C**
