# Supplementary material for: Growth Substrate and Prophage Induction Collectively Influence Metabolite and Lipid Profiles in a Marine Bacterium
Source: mSystems. 2022 Aug 16;7(5):e00585-22. doi: 10.1128/msystems.00585-22 (PMC9600351; doi:10.1128/msystems.00585-22)
Supplement: TABLE S3 [file msystems.00585-22-s0006.docx]

**Table S3.** Optical density and viable counts data for strains CB-A and CB-D. These tables are shown for all substrates for cells grown in **(A)** complex (SMM), **(B)** glutamate, and **(C)** acetate media. Averages and standard deviations data for biological replicates are reported for all treatments and time points (n= 3 for complex grown cells; n = 5 in glutamate and acetate grown cells).


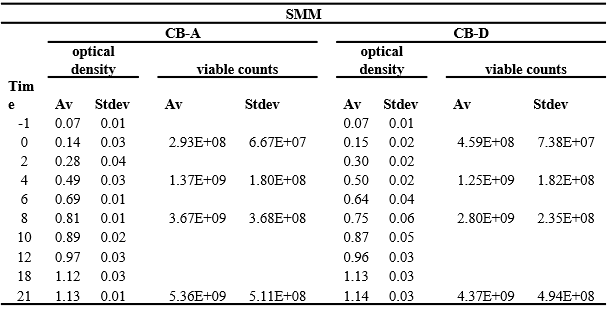

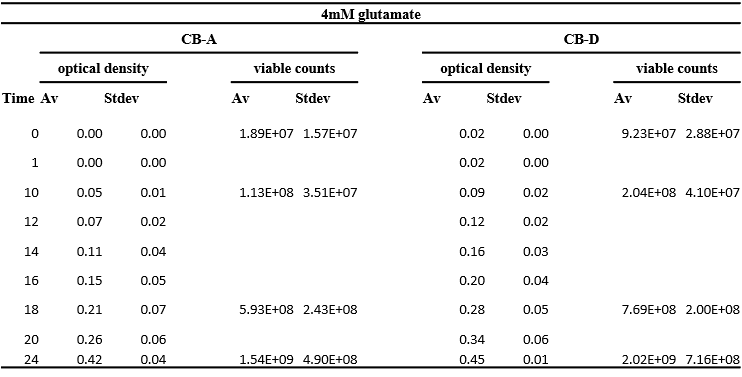

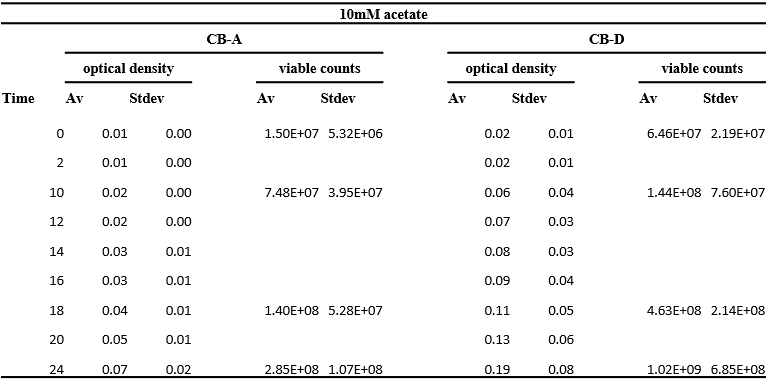


**A**

**B**

**C**
