# Supplementary figures and images for: Growth Substrate and Prophage Induction Collectively Influence Metabolite and Lipid Profiles in a Marine Bacterium
Source: mSystems. 2022 Aug 16;7(5):e00585-22. doi: 10.1128/msystems.00585-22 (PMC9600351; doi:10.1128/msystems.00585-22)

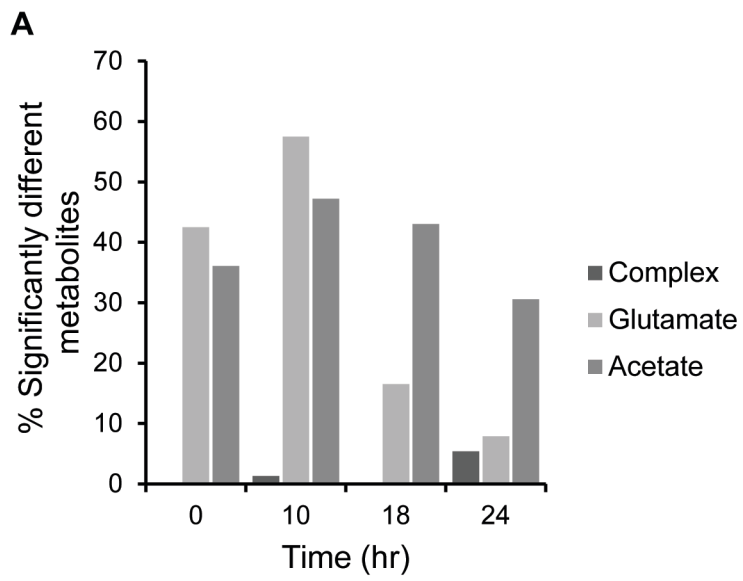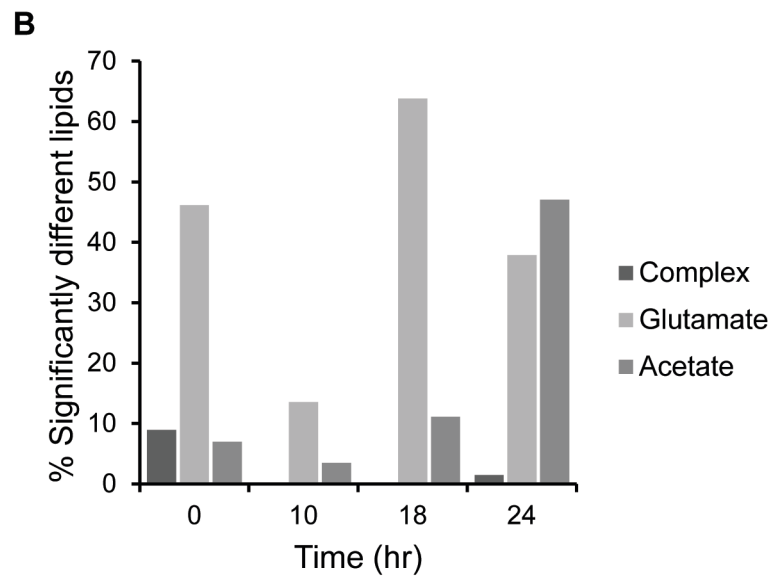

\*Complex time points were as follows: t=0 hrs, t=4 hrs, t=8 hrs, and t=21 hrs

Supplement: FIG S1 [file msystems.00585-22-s0001.pdf]

|           | <i>Sulfitobacter</i> sp. CB-D                                                       |                                                                                     |                                                                                     |                                                                                     | <i>Sulfitobacter</i> sp. CB-A                                                       |                                                                                     |                                                                                       |                                                                                       |
|-----------|-------------------------------------------------------------------------------------|-------------------------------------------------------------------------------------|-------------------------------------------------------------------------------------|-------------------------------------------------------------------------------------|-------------------------------------------------------------------------------------|-------------------------------------------------------------------------------------|---------------------------------------------------------------------------------------|---------------------------------------------------------------------------------------|
|           | T0                                                                                  | T4/10                                                                               | T8/18                                                                               | T21/24                                                                              | T0                                                                                  | T4/10                                                                               | T8/18                                                                                 | T21/24                                                                                |
| Complex   | 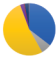  | 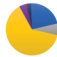  | 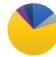  | 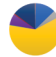  | 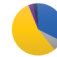  | 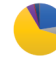  | 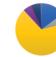  | 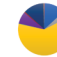  |
| Glutamate | 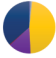 | 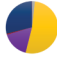 | 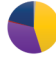 | 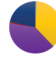 | 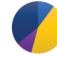 | 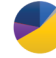 | 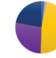 | 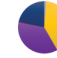 |
| Acetate   | 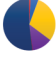 | 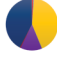 | 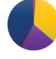 | 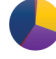 | 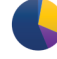 | 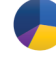 | 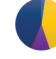 | 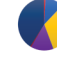 |

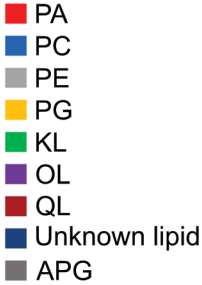

Supplement: FIG S3 [file msystems.00585-22-s0003.pdf]

**Lysine lipid**

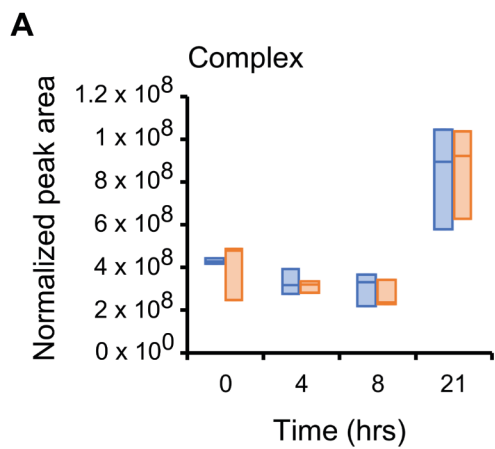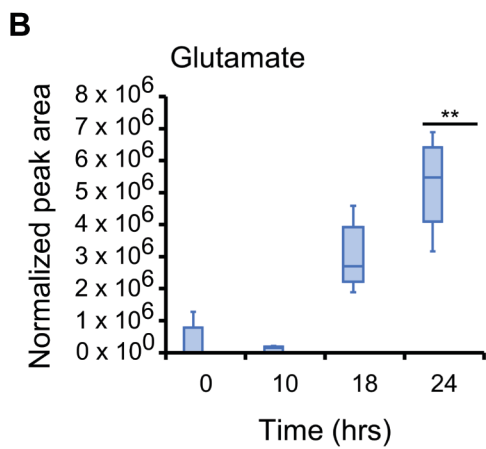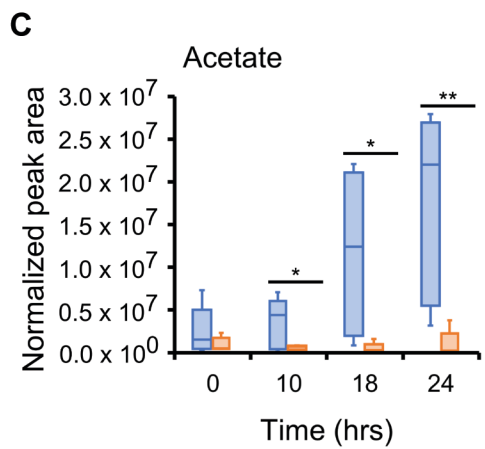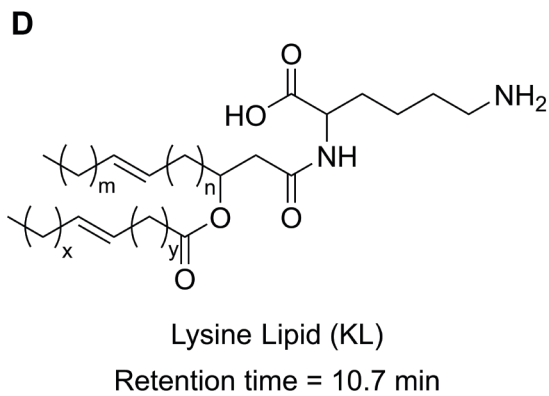

Supplement: FIG S2 [file msystems.00585-22-s0002.pdf]
